# Superabsorbent Polymer Seed Coating Reduces Leaching of Fungicide but Does Not Alter Their Effectiveness in Suppressing Pathogen Infestation

**DOI:** 10.3390/polym14010076

**Published:** 2021-12-26

**Authors:** Marcela Gubišová, Martina Hudcovicová, Pavel Matušinský, Katarína Ondreičková, Lenka Klčová, Jozef Gubiš

**Affiliations:** 1 National Agricultural and Food Centre, Research Institute of Plant Production, Bratislavská cesta 122, 921 68 Piestany, Slovakia; marcela.gubisova@nppc.sk (M.G.); martina.hudcovicova@nppc.sk (M.H.); lenka.klcova@nppc.sk (L.K.); jozef.gubis@nppc.sk (J.G.); 2 Department of Botany, Faculty of Science, Palacký University in Olomouc, Šlechtitelů 27, 783 71 Olomouc, Czech Republic; pavel.matusinsky@upol.cz or; 3 Department of Plant Pathology, Agrotest Fyto, Ltd., Havlíčkova 2787, 767 01 Kromeriz, Czech Republic

**Keywords:** crop production, drought, seed dressing, seed coating, superabsorbent polymers

## Abstract

Superabsorbent polymers (SAPs) applied to soil have been recognized as water reservoirs that allow plants to cope with periods of drought. Their application as a seed coat makes water available directly to the seeds during their germination and early growth phase, but on the other hand, it can affect the efficiency of plant protection substances used in seed dressing. In our experiments, we evaluated the effect of seed coating with SAP on fungicide leaching and changes in their effectiveness in suppressing *Fusarium culmorum* infestation. Leaching of fungicide from wheat seeds coated with SAP after fungicide dressing, as measured by the inhibition test of mycelium growth under in vitro conditions, was reduced by 14.2–15.8% compared to seeds without SAP coating. Germination of maize seeds and growth of juvenile plants in artificially infected soil did not differ significantly between seeds dressed with fungicide alone and seeds treated with SAP and fungicide. In addition, plants from the seeds coated with SAP alone grew significantly better compared to untreated seeds. Real-time PCR also confirmed this trend by measuring the amount of pathogen DNA in plant tissue. Winter wheat was less tolerant to *F. culmorum* infection and without fungicide dressing, the seeds were unable to germinate under strong pathogen attack. In the case of milder infection, similar results were observed as in the case of maize seeds.

## 1. Introduction

Seed germination and early development of healthy juvenile plants are limiting factors for the establishment of crop yields [1]. In agricultural praxis, it is common to use pesticides to protect germinating seeds and juvenile plants during the early growth phase in order to avoid damage by insect pests and plant pathogens [2]. The seeds of many crops are routinely dressed with fungicides and insecticides before sowing. Nowadays, farmers also have to deal with another task. Global climate change brings the problem of deepening drought even in locations where it was not typical before. Drought is a major abiotic stress that threatens the efficiency of agricultural production because seed germination, plant growth, and fruiting in drought conditions are reduced [3,4]. Tackling this problem will be crucial for crop production in the future. For 2100, the FAO predicted that climate change with higher temperatures, droughts, floods, and irregular rainfall would lead to a decline in the production of major cereals (20–45% for maize, 5–50% for wheat, and 20–30% for rice), which endangers food security [5].

The growing world population and a shortage of water for irrigation availability motivates people look for new ways to efficiently use water in the agricultural sector. In addition to crop breeding for drought resistance [6] and changes in soil management [7], there is the possibility of using superabsorbent polymers (SAPs) that are able to absorb and retain 300 to 500 times more water than their own mass [8]. Such polymers applied to the soil serve as mini reservoirs of water, from which water is removed upon the root demand through an osmotic pressure difference [9]. This approach allows for improving seed germination, root growth during drought stress, and transplantation stress [8]. Moreover, smart (stimuli-based) polymer capsules with the controlled release of substances can be used to protect plants against pathogens or to apply micronutrients when plants need them [10]. Such intelligent supplies with slow or controlled release systems, as well as the use of polymers as biosorbents adsorbing pollutants from the environment, are also interesting from an ecological point of view [11]. 

The application of SAPs as a hydrogel in soil started in 80th and from then, several types of polymers (polyacrylamide, polyacrylate, starch, cellulose, alginate, or other polysaccharides) have been tested to improve water retention capacity and biodegradability [11]. SAPs, which are usually added as granulated or powder conditioner into the soil, modifies the water retention capacity and enhances plant growth [12,13,14,15,16,17]. Seed-coating technology is a modern technology that allows the delivery of a wide range of active compounds directly onto the seeds and streamlines their utilization by plants. Moreover, this technology enables to minimize the amount of applied SAP compared to the addition as a soil supplement. In seed coating technology, synthetic polymers are commonly used [18,19] due to their suitable chemical properties, but natural polymers such as hydroxypropyl methylcellulose [20], starch [21], or chitosan with alginate [22] have also been tested. The application of SAPs as swelling hydrogels on the seed surface creates a direct line of available water for seeds and can help plants cope with water shortage during the germination and early growth phase. It can help establish vegetation more efficiently and produce higher yield. Su et al. [19] evaluated the effect of five SAPs: Polyacrylamide, sodium polyacrylate, and three commercial products on the germination of legume seeds of *Caragana korshinskii* and the physiological parameters of plants in drought conditions. They observed the positive effect of these SAPs on the percentage and energy of seed germination. All measured parameters of physiological stress (the relative electrical conductivity, proline, malondialdehyde, H_2_O_2_ content, and peroxidase activity) were reduced. Jarecki and Wietecha [22] observed a 0.5-t ha^−1^ higher yield of soybean from coated seeds in unfavorable conditions. In the experiments of Pačuta et al. [23], the synthetic polymer Aquaholder^®^Seed, the same as in our experiment, was used to treat sugar beet seeds and the positive effect of SAP on the leaf area index, root yield, and sugar yield was confirmed. 

Coating of seeds with polymers is considered an innovative tool to cope with the lack of water during seed germination, but on the other hand, it can affect the effectiveness of plant protection substances used in seed dressing. It was also shown in previous studies on sand matrices that polymer seed coating can reduce the leaching of pesticides from the seeds surface [24,25]. However, this process in real conditions is affected by physicochemical properties of the soil, including organic matter content, pH, humidity, and it depends also on pesticide characteristics, especially solubility [26]. Retainment of pesticides by polymer is also controlled by its molecular weight and chemical structure (polarity, branching, and side chain length) [27]. Therefore, further studies are needed to address this issue for SAP-coated seeds.

There is a lack of publications on changes in pesticide efficacy against soilborne pathogens in polymer-coated seeds. Some papers have been published on infestation by storage fungi on seeds treated with polymer, fungicide, or their combination. Kumar et al. [28] compared seed coating with 12 polymers-based and 1 clay-based seed coatings with fungicide treatment or untreated seeds and observed statistically lower fungal infection in seeds coated with the most polymers after 6 months of storage, but higher compared to fungicide treatment. Similar experiments were performed with cotton [29] or chili [30] where seeds were treated with undefined polymer, fungicide thiram, or their combination and stored in a polymer bag or aluminum foil. The lowest infection of seeds by storage fungi in a combined treatment of polymer with fungicide was observed. 

The aim of our work was to measure the effect of seed coating with SAP on the efficiency of fungicides contained in seed dressing and to evaluate the effect of seed coating with SAP on the leaching of fungicides. We hypothesized that: (1) SAP coating will affect the release of pesticides from seed dressing, but (2) it will not affect the effectiveness of fungicide dressing in suppressing pathogen infestation.

To achieve these goals, *Fusarium culmorum*, a serious pathogen of maize and winter wheat belonging to the major hosts of this pathogen was used. *Fusarium culmorum* was chosen due to its severity and the ability of rapid mycelial growth [31]. This makes it suitable for short-term tests in the seed germination phase, which was the goal of our experiment. *F. culmorum* attacks a wide range of plant species, especially cereals, causing a decrease in crop yield and grain quality with contamination by mycotoxins. The fungus spreads by soil, air, and infected seeds, and causes diseases known as fusarium foot and root rot leading to the destruction of seedlings, and fusarium head blight damaging kernels and spikes [32]. In phytopathology, methods of artificial inoculations are generally employed. To evaluate the tolerance of host genotypes to fusarium seedling blight, the root-dip inoculation method is usually used [33]. In some cases, the methods of soil contamination are more appropriate. This can be done by soil irrigation with a spore suspension [31], or by adding a substrate overgrown by fungal mycelium to the soil [34]. In our experiments, the second method was used, and the fungal inoculum was applied to the soil on a natural carrier, thus simulating the contamination of the soil with post-harvest residues.

## 2. Materials and Methods

### 2.1. Biological Material

Seeds of maize (*Zea mays* L.) cv. Celong were dressed with fungicide Redigo M (Bayer S.A.S., Lyon, France) with active substances of prothioconazole 100 g L^−1^ + metalaxyl-M 20 g L^−1^ in a dose of 15 mL per sowing unit (50,000 seeds), wheat (*Triticum aestivum* L.) seeds cv. Luneta were dressed with Redigo Pro (Bayer S.A.S., Lyon, France) with active substances of prothioconazole 150 g L^−1^ + tebuconazole 20 g L^−1^, in a dose of 0.5 L t^−1^. Seeds without or after dressing with fungicides were coated with SAP (Aquaholder^®^Seed, PeWaS s.r.o., Bratislava, Slovak Republic, patent appl. no. PCT/SK2019/050008) based on acrylic acid, potassium salt of acrylic acid, and acrylamide.

Seed variants:

1. Control seeds without any treatment (NT);
2. Seeds coated with SAP (S);
3. Seeds dressed with pesticides (P);
4. Seeds dressed with pesticides and coated with SAP (PS).

The strain of *Fusarium culmorum* (W.G. Sm.) Sacc., tribe VURV-F 494 (Collection of Agriculturally Important Fungi, Crop Research Institute, Prague-Ruzyně, Czech Republic) used had shown a high level of virulence and strong production of deoxynivalenol (DON) as described in our previous study [35]. Mycelium was freshly grown in Petri dishes with potato dextrose agar (PDA; Duchefa Biochemie, Haarlem, The Netherlands) at 22 °C for 7 or 28 days in the dark for its use as an inoculum. 

### 2.2. In Vitro Test of Mycelium Growth Inhibition

For this test, a modified agar diffusion method with spot inoculation was used that enables the determination of antimicrobial activity of different substances [36]. Petri dishes (ø 90 mm) with PDA were inoculated with the 7-day-old culture of *F. culmorum* using mycelium plugs 7 mm in diameter placed in the center of each dish. Two days later, eight wheat seeds of treatments NT, P, and PS were evenly laid on a circle near the edge of each dish. The diameter of the fungal colony was measured on the 7th and 10th day of cultivation at 22 °C in the dark. The fungal colony diameter was determined as an average of minimal and maximal diameter of the colony. The inhibition zone was calculated as: IZ = C − T (C = diameter of fungal mycelium of control variant, T = diameter of fungal mycelium of treated variants), from which the percentage of mycelial growth inhibition was calculated as: MGI = (IZ/C) × 100% [36]. The experiment was repeated twice with 10 plates for each treatment. 

### 2.3. Effect of Combined Seed Treatment with Pesticides and SAP on the Seed Germination in Soil Contaminated with F. culmorum

For soil inoculation, a modified methodology of Koch et al. [34] was used. Sporulating mycelium from 28-day-old culture was scraped off the PDA medium and transferred into flasks containing 100 mL of sterilized distilled water. The conidial suspension was adjusted to 10^5^ conidia mL^−1^. Two Erlenmeyer flasks with 40 mL of autoclaved (121 °C, 20 min.) seeds of millet (inoculation I), pearl barley (inoculation II), or barley groats (inoculation III) were inoculated with 12 mL of conidial suspension. Infected material regrew by fungal mycelium in 3 days at 22 °C in the dark. The potting substrate was mixed from horticulture substrate (Klasmann-TS 3; Klasmann-Deilmann, Geeste, Germany), sand, and arable soil (1:1:1) and sterilized by autoclaving (121 °C, 20 min.). Inoculations I and II were done by adding millet seeds (I) or pearl barley (II) overgrown by fungal mycelium in the amount of 2% (V/V) to the potting substrate and carefully mixed. For inoculation III, which was performed in the next experiment due to the devasting effects of I and II inoculation on wheat seed without pesticide treatment, two pieces of barley groats were added to the substrate in the center of the pots (in the same depth as the seeds were placed). 

Six seeds of maize or 10 seeds of wheat were sown in each pot (200-mL plastic cups for wheat or 300-mL cups for maize). In the case of inoculation III, the seeds were sown on a circle near the edge of 200 mL pots. The substrate was watered with 20/30 mL of tap water and cultivated at 18–20 °C under 16-h photoperiod (light intensity 200 µmol m^−2^ s^−1^) covered with a plastic cover to maintain humidity. The pots were watered again after five days with a half dose of water. All seed treatments of wheat and maize seeds (NT, S, P, and PS) were used in these experiments, three pots were used for each variant. After 7 days, germination percentage and the length of shoots were measured. On the 10th day, young leaves were taken for DNA isolation.

### 2.4. Quantitative Analysis of Fusarium DNA in Juvenile Plants

Approximately 0.1 g of *F. culmorum* mycelium was harvested from Petri dishes and 0.1 g of young maize and wheat leaves was cut from each pot (mixed sample of all plants in a pot). Samples were ground to a fine powder in a cooled mortar using liquid nitrogen, homogenized, and pure total genomic DNA was extracted using the DNeasy Plant Mini Kit (Qiagen, Hilden, Germany). The concentration and quality of isolated DNA were checked spectrophotometrically using NanoDrop1000 Spectrophotometer (Thermo Fisher scientific Inc., Waltham, MA, USA) and a total DNA was quantified by Qubit™ Flex Fluorometer (Invitrogen™, Thermo Fisher Scientific Inc., Waltham, MA, USA).

Quantification of pathogen DNA in wheat and maize leaves was carried out by a real time-PCR method using ABI PRISM^®^ 7000 machine (Applied Biosystems, Foster City, CA, USA) in MicroAmp optical 96-well plates (Applied Biosystems, Foster City, CA, USA). TaqManTM probe FC92s1 5′FAM 3′MGB and primer pair Fc92s1 specific for *F. culmorum* were used according to Leišová et al. [37]. Reaction was carried out in 25-µL reaction volume consisting of 12.5-µL TaqMan Universal PCR Master Mix (Applied Biosystems, Foster City, CA, USA), 300 nM of each primer, 200 nM of TaqMan MGB probe (labelled with FAM fluorescent dye), and 50 ng of total DNA. Conditions of PCR were as follows: 95 °C for 10 min., 40 cycles: 95 °C for 15 s and 60 °C for 1 min.

As standards for standard curves five dilutions of pure *F. culmorum* DNA (1, 10, 100, 1000, and 10,000 pg µL^−1^) were applied in triplicate in every run. For an evaluation of the results, the ABI PRISM^®^ 7000 software (Applied Biosystems, Foster City, CA, USA) was used, in which unknown samples are quantified from measured CT (cycle threshold) values by interpolation using the regression equation derived from standard curves.

### 2.5. Statistical Analysis

Experimental data were analyzed by ANOVA and the means were separated by the LSD test (the least significant difference) at α = 0.05 using the statistical software STATGRAPHICS XVII–X64 (Statpoint Technologies, Inc., Warrenton, VA, USA).

## 3. Results 

### 3.1. In Vitro Test of Mycelium Growth Inhibition

**Figure 1:**
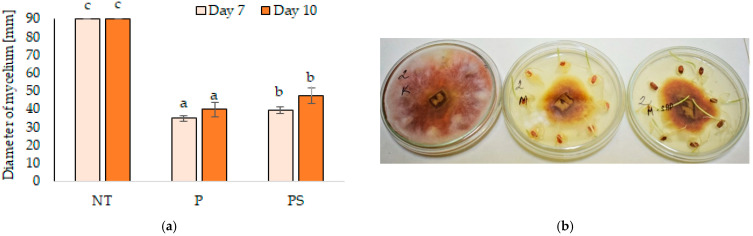
Inhibition of (*F. culmorum*) mycelial growth by seed dressing with pesticides combined with or without SAP coating. (**a**) Inhibition of fungal growth expressed by the fungal mycelium diameter (NT–control seeds, P–seeds dressed with pesticides, and PS–seeds dressed with pesticides and coated with SAP). The errors of the mean values are expressed as standard deviation (± SD). Different letters indicate statistically significant differences by LSD test (α = 0.05), a statistical test was done separately for each term. (**b**) Growth of fungal mycelium (*F. culmorum*) on plates with wheat seeds, from left: Control–not treated seeds, seeds dressed with pesticides, and seeds dressed with pesticides and coated with SAP.

In our experiment, it was discovered that mycelium growth in Petri dish with a nutrient medium had been reduced stronger using the seeds without SAP. After 7 days, plates with control seeds without any treatment were overgrown with fungal mycelium on the whole area, while inhibition of mycelial growth was observed on the plates with treated seeds (Figure 1). The diameter of fungal mycelium was larger for seeds with a combined treatment. The MGI on the plates with the pesticide-treated seeds reached 61.2%, compared to 56.2% on the plates with seeds coated with SAP after pesticide dressing. Measurement on the 10th day confirmed the same trend and the percentage of mycelial growth inhibition reached 55.7% and 47.4%, respectively. 

### 3.2. Effect of Combined Seed Treatment with Pesticides and SAP on Seed Germination in Soil Contaminated with F. culmorum 

Seed germination of wheat and maize was affected by the presence of the fungal pathogen in the soil. Soil contamination had a more detrimental effect on wheat seeds, where seeds not dressed with pesticides did not germinate after inoculation I and II regardless of the SAP coating (Table 1). The germination percentage of seeds treated with pesticides was not statistically different in control or contaminated soil. Seeds treated with pesticides in combination with SAP germinated in a lower percentage than the seeds treated only with pesticides in the case of strong soil infestation (inoculation I and II), but there was no statistical difference between these seed variants in the case of weaker soil contamination (inoculation III). All variants of maize seeds germinated in all types of fungal inoculation. Germination of control (NT) seeds was significantly affected by soil contamination, but seeds coated with SAP only germinated significantly better in comparison with control seeds. The germination percentage of seeds treated with pesticides was not significantly affected by fungal contamination in the soil as well as seeds with a combined treatment of pesticides and SAP (Table 1).

**Figure 2:**
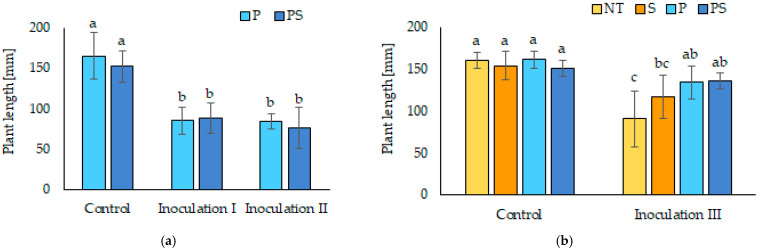
The length of wheat plants in the control soil or soil contaminated with *F. culmorum*; (**a**) inoculation I and II, (**b**) inoculation III measured 7 days after sowing (NT–not treated seeds, S–seeds coated with SAP, P–seeds dressed with pesticides, and PS–seeds dressed with pesticides and coated with SAP). The errors of the mean values are expressed as standard deviation (±SD). Different letters indicate statistically significant differences by LSD test (α = 0.05).

In addition, the growth of juvenile plants in the soil substrate contaminated with *F. culmorum* was strongly affected. In the case of inoculation I and II, seeds of wheat not treated with pesticides did not germinate (variants NT and S). Growth of plants from the seeds treated with pesticides alone did not differ significantly from the seeds treated with pesticides and SAP. The growth of plants in contaminated soil was slower than in the control soil and their height reached only 50–58% (see Figure 2a). The weaker inoculation III enabled the growth of plants from wheat seeds without pesticides. Plants from not-treated seeds grew significantly slower than from seeds with pesticides (P and PS), and there was no statistical difference between seeds treated with pesticides only or with pesticides + SAP. Plants from SAP-coated seeds (S) grew in contaminated soil 1.3 times faster than not-treated seeds and 1.15 times slower than variants with pesticides (P and PS), but the differences were not statistically significant. The only statistically significant difference was observed between pesticide treatments (P or PS) and control seeds (see Figure 2b). 

**Figure 3:**
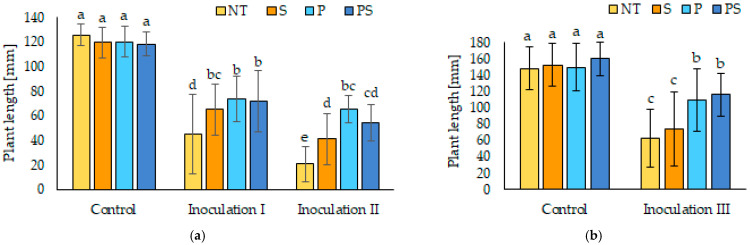
The length of maize plants in the control soil or soil contaminated with *F. culmorum*; (**a**) inoculation I and II, (**b**) inoculation III measured 7 days after sowing (NT–not treated seeds, S–seeds coated with SAP, P–seeds dressed with pesticides, and PS–seeds dressed with pesticides and coated with SAP). The errors of the mean values are expressed as standard deviation (±SD). Different letters indicate statistically significant differences by LSD test (α = 0.05).

The growth of maize plants in the soil substrate contaminated by *F. culmorum* was also strongly affected. A part of seeds was able to germinate also without pesticide dressing in all types of inoculation, but the growth of plants from NT seeds was significantly slower compared to other seed variants. After inoculation II, the differences among seed variants were the most significant (see Figure 3a). Seeds coated with SAP alone grew slower than P or PS variants, but the statistically significant difference was observed only between variants S and P in the case of inoculation II. Slightly slower growth of plants from seeds with combined treatment (PS) was observed compared to the P variant for inoculation II, but the difference was not statistically significant. The height of plants from NT seeds reached only 16.4 or 35.9% (inoculation II or I respectively) of plants in the control soil, while the height of plants from SAP-coated seeds reached 34.4 or 54.6%, and for pesticide-treated seeds (P or PS) it was 54.7–61.5% compared to plants in the control soil. For inoculation III, the differences among seed variants were similar to inoculation I, but the differences between NT and S variants were not statistically significant (see Figure 3b). 

### 3.3. Quantitative Analysis of Fusarium DNA in Juvenile Plants

**Figure 4:**
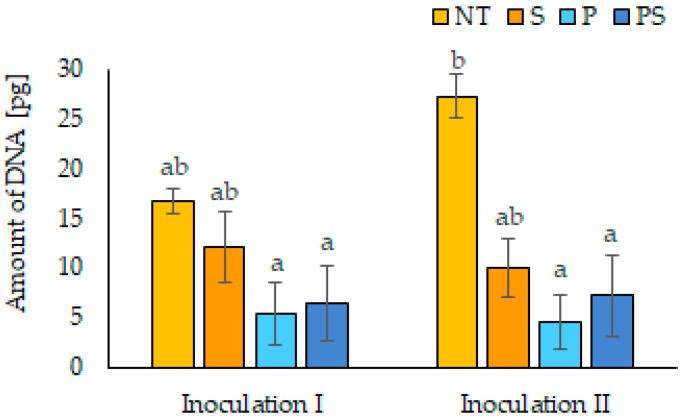
The amount of *Fusarium* DNA in plant tissue of maize juvenile plants grown in the contaminated soil in pg per 50 ng of total DNA. (NT–not treated seeds, S–seeds coated with SAP, P–seeds dressed with pesticides, and PS–seeds dressed with pesticides and coated with SAP). The errors of the mean values are expressed as standard deviation (±SD). Different letters indicate statistically significant differences by LSD test (α = 0.05).

The amount of pathogen DNA in plant tissue was measured by real-time PCR. The highest amount of the pathogen DNA was determined in maize plants from NT seeds in inoculation II. In both inoculation I and II, the amount of pathogen DNA in plant tissue decreased in the order of NT seeds, SAP-coated seeds, and the combined treatment of pesticides + SAP to pesticide-dressed seeds (see Figure 4). The percentage of pathogen DNA reached from 0.09–0.11 × 10^−3^ of the total DNA isolated from tested plants for pesticide-dressed seeds to 0.33–0.54 × 10^−3^ for NT seeds. In the case of inoculation III, the pathogen DNA was under the detection limit in both, wheat and maize plants. Due to this reason and the inability of wheat seeds not being treated with pesticide to germinate under the pathogen pressure in the case of inoculation I and II, it was not possible to compare the amount of pathogen among the wheat treatments.

## 4. Discussion

SAPs form water-insoluble polymer 3D networks with a high-water holding capacity. Due to this capability, SAPs are used for a variety of purposes including medical or personal care, food packaging, oil drilling, wastewater treatment, and others. In the agriculture sector, SAPs have been recognized as soil conditioners that increase water retention capacity and form water reservoirs around the plant roots [38]. Although mostly used as a soil supplement in the form of granules or powder, their targeted application directly onto the seeds forms a hydrogel bed around the seeds, which helps them germinate in periods of water shortage and enables more efficient stand establishment [39]. On the other side, leaching and the activity of substances for crop protection against pests and diseases dressed on the seeds could be changed. 

The effect of SAP coating on fungicide leaching was measured indirectly–by the inhibition test of mycelium growth under in vitro conditions. The chemicals used in seed dressing are expected to diffuse spontaneously into the nutrient medium and form a barrier for mycelial growth. Seeds treated with fungicide and placed on a circle near the edge of the dish slowed down the growth of fungal mycelium from the centre of the Petri dish. The inhibition zone was larger in the case of seed treated with fungicide alone. These results may support the hypothesis that SAP coating on seeds could reduce the release of pesticides from the seed surface probably by creating a barrier for leaching substances applied to the seed surface. However, this process in real soil conditions is affected by the properties of soil, polymer, and pesticides [26,27]. Such physical or chemical principles of polymers interaction with other chemical substances predetermine them as a device for the controlled release of substances affecting plant growth and health [10]. Lower leaching and mobility of pesticides leads to the reduction of their phytotoxicity and environmental pollution [9,10]. This may apply not only to the encapsulated substances themselves but also to seeds coated with pesticides and superabsorbents. In the experiment of Ludwig et al. [24], the effectiveness of seed coating with polymer Likoseed Vermelho^®^ applied at a dose of 1 mL kg^−1^ of soybean seed in reducing the pesticide losses was quantified. The polymer was applied by two methods–as a mix with pesticides or after pesticides treatment. The seeds were sown in the sand, which was then saturated at 100% water holding capacity. Quantity of insecticide Thiamethoxam in leached solution was measured by the method of ultra-high performance liquid chromatography-tandem mass spectrometry and polymer application was found to reduce losses of substances by 20% regardless of the method of application. The same group [25] performed a similar experiment with rice seed treated with combined pesticide treatment (insecticide Thiamethoxam + fungicide Metalaxyl-M) and one of four tested commercial polymers (Florite polymers 1127^®^, Solid Resin GV5^®^, Polyseed CF^®^, and VermDynaseed^®^) and confirmed reduced insecticide leaching in three of them. They also evaluated the percentage of fungi (*Fusarium* sp., *Penicillium* sp., *Aspergillus* sp., and *Drechslera* sp.) present on treated rice seeds and did not find statistically significant differences between seeds treated with pesticides alone and combined treatments with pesticides + polymer. Avelar et al. [40] studied the leaching of the insecticide Furazin, which was applied on corn seeds together with polymer Polyseed CF. After sowing seeds in sand and irrigation, minimized leaching of insecticide was shown by measuring the amount of leached zinc (a component of Furazin) in eluate by atomic absorption spectrophotometry.

After sowing the seeds in soil contaminated by fungal inoculum of *Fusarium culmorum* we found out that maize seeds were more tolerant to the contamination of soil by *Fusarium* compared to winter wheat, whose fungicide-untreated seeds were unable to germinate under the strong pressure of a pathogen induced by inoculation I (infected millet seeds) and II (pearl barley). These two inoculations proved to be too strong to comprehensively assess the impact of SAP coating on wheat seeds. Only the weaker inoculation III by barley groats, where an approximate 20-fold lower amount of inoculum carrier was applied, allowed the seeds without the fungicide treatment to germinate. 

A difference in the pathogen’s attack strength between inoculation I and II was also observed, and contamination of soil by pearl barley proved to be more severe. This is in the contrary with the results of Koch et al. [34], who observed a stronger pathogen attack using infected millet seeds. However, this variation could be due to the use of *F. culmorum* strain with a different aggressiveness level and barley groats of a different size category. More importantly was the observation that there were no significant differences found in seed germination and seedlings growth of wheat (inoculation III) and maize between pesticide treatment and combined treatment of seeds with pesticides and SAP. In the case of strong soil contamination (I and II), wheat seeds treated with pesticides and SAP germinated in a lower percentage than the seeds treated with pesticides alone. Only in this one case was a negative effect of SAP on the effectiveness of fungicides observed, although the length of plants was not affected. This soil infection was set too strong and, moreover, with a very aggressive strain of the pathogen because the soil was visibly overgrown by fungal hyphae after one week. However, such a situation is not expected in natural conditions, or only in very rare cases. In addition, in the case of maize seeds, the SAP coating alone protected the germinating seeds from a pathogen attack and the seedling length was statistically higher compared to untreated seeds, while statistically not different from the seeds dressed with pesticides. For winter wheat seeds, the length of seedlings from SAP-coated seeds after inoculation III was also higher, but did not differ statistically from untreated seeds. Overall, pesticide seed dressing protected germinating seeds from a severe attack of *F. culmorum* regardless of the SAP coating, but SAP coating alone was also able to reduce infection, albeit to a lesser extent. These observations were also confirmed by the quantitative analysis of pathogen DNA in plant tissue of maize seedlings. Real-time PCR is very sensitive to detecting the contamination of plants, although symptoms of the disease are not yet visible. Even though the amount of pathogen DNA was low and statistical significance was observed only between untreated seeds and other variants after inoculation II, the trend of decreasing the amount of the pathogen DNA in order from untreated seeds to the pesticide-treated seeds corresponds with growth retardation of juvenile plants of maize in the same order. 

The positive effect of SAP seed coating on the weakening of pathogen attack opens possibilities to replace chemical seed treatments by environmentally more friendly methods or combining polymers with biopesticides, mycorrhizal fungi, or other bioactive substances to enhance plant health and vitality. Seed coating is one of the most cost-effective technologies to deliver bioactive substances or beneficial microorganisms for plant disease control [41]. This technology avoids the use of large amounts of inoculant for successful colonization and is economically more feasible compared to direct application into the soil [42]. For seed coating, natural or synthetic hydrogels can be used as carriers for microorganisms or biological substances. Such an approach was chosen in the experiment of Elshafie et al. [41] who studied the effect of oregano essential oil and ornithine lipid in a hydrogel seed coating based on gelatin, PEG, and starch on fungal diseases of *Phaseolus vulgaris* (*Fusarium oxysporum*, *Sclerotinia sclerotiorum*, *Aspergillus flavus*, *Rhizoctonia solani*) and observed significant fungal inhibition. In the work of Kimmelshue et al. [43], coating of maize seeds with bacteriophages CN8 in polyvinyl polymer reduced the infection of seeds and germinating plants by *Clavibacter michiganensis* subsp. *Nebraskensis*, the causal agent of Goss’s wilt. 

In our experiment, the copolymer of acrylic acid, the potassium salt of acrylic acid, and acrylamide was used for seed coating. In soil, polyacrylamide can be decomposed by photolytic degradation with UV light or by biodegradation by bacteria [44,45]. Acrylamide is considered a neurotoxin and a potential carcinogen. Several studies suggest that naturally occurring microbes in soils are able to degrade acrylamide to the nontoxic products ammonia and acrylic acid [44]. It has been shown that microorganisms utilize not only acrylamide but also polyacrylamide and its derivatives as a source of nitrogen and/or carbon under aerobic as well as anaerobic conditions [46]. However, the potential risks of using acrylamide derivates must be monitored. 

Based on this information, we can assume that the polymer used for seed treatments in our experiments will eventually decomposes into CO_2_, water, ammonia, nitrogen, and potassium salt, i.e., substances that are naturally found in soil. Oksińska et al. [47] identified two bacteria, *Rhizobium radiobacter* 28SG and *Bacillus aryabhattai* 31SG, isolated from the watered SAP, that were able to biodegrade up to 36% of the similar copolymer of acrylamide and potassium acrylate containing 5.28% of unpolymerized monomers in 30 days. Despite the expected biodegradability, there are efforts to replace synthetic SAPs with natural ones such as starch [21], cellulose [14], chitosan or alginate [22], or with synthetic-biopolymer hybrid SAPs that combine the desired chemical properties of synthetic polymers–such as mechanical strength, high water capacity, and stability, and lower cost and biological safety of natural polymers [48]. Proteins are also currently gaining a lot of attention as a replacement for synthetic SAPs because they are biodegradable and available from agricultural waste [49,50]. In addition, they themselves act as biostimulants or may be supplemented with nutrients that can be released in a controlled manner [51]. While most studies on SAP have been focused on improving their water retention capacity, their potential role in plant protection improves the prospects for large-scale applications in agricultural praxis.

## 5. Conclusions

Seed coating with SAPs that enables better germination and more effective crop establishment during periods of drought could alter the effectiveness of substances for crop protection used in seed dressing. In our experiments, four variants of wheat and maize seed treatment were used, with or without fungicide dressing combined with or without SAP. SAP coating in variants with fungicides was applied after fungicide dressing. Our results may support the hypothesis concerning the leaching of fungicides and their effectiveness in suppressing pathogen infestation in SAP-coated seeds. In the in vitro test of mycelial growth inhibition, a positive effect of SAP on the reduction of fungicide leaching from seeds into the environment was observed. Germination of seeds and growth of juvenile plants of winter wheat and maize in soil contaminated with fungal pathogen *Fusarium culmorum* were significantly better for fungicide-treated seeds, but SAP alone provided partial protection for seeds during the germination and early growth phase. In addition, the statistically significant negative effect of SAP seed coating on the effectiveness of the fungicides and the growth of juvenile plants of maize was not observed. Wheat seeds treated with pesticides in combination with SAP germinated at a lower percentage than seeds treated only with pesticides in the case of strong soil contamination. After weaker contamination, the germination of fungicide treated seeds were not affected by SAP. The length of juvenile plants of wheat was not statistically different between variants with SAP and SAP with fungicide in all variants of contamination. 

## Figures and Tables

**Table 1:** Germination percentage of non-treated (NT) maize and wheat seeds and seeds treated with SAP (S), pesticide dressing (P), or pesticide with SAP (PS) in the control soil or soil contaminated with the inoculum of *F. culmorum*. The errors of the mean values are expressed as standard deviation (±SD). Different letters indicate statistically significant differences by LSD test (α = 0.05).

Seed Treatment	NT	S	P	PS
**Inoculation**	**Maize**			
Control	91.7 ± 16.7 ^abc^	91.7 ± 9.6 ^abc^	100 ± 0.0 ^a^	95.8 ± 8.3 ^ab^
Inoculation I	66.7 ± 16.7 ^ef^	77.8 ± 9.6 ^cde^	100 ± 0.0 ^a^	100 ± 0.0 ^a^
Inoculation II	55.6 ± 9.6 ^f^	88.9 ± 9.6 ^abc^	94.4 ± 9.6 ^ab^	88.9 ± 9.62 ^abc^
Inoculation III	66.7 ± 16.7 ^ef^	72.2 ± 9.6 ^de^	94.4 ± 9.6 ^ab^	100 ± 0.0 ^a^
	**Wheat**			
Control	85.0 ± 5.7 ^a^	85.0 ± 10.0 ^a^	90.0 ± 14.1 ^a^	82.5 ± 5.0 ^ab^
Inoculation I	0.0 ^g^	0.0 ^g^	90.0 ± 10.0 ^a^	63.3 ± 11.6 ^cd^
Inoculation II	0.0 ^g^	0.0 ^g^	93.3 ± 5.8 ^a^	66.7 ± 15.3 ^bcd^
Inoculation III	50.0 ± 20.0 ^d^	76.7 ± 15.3 ^abc^	83.3 ± 11.6 ^ab^	86.7 ± 5.8 ^a^
